# A tandem-repeat dimeric RBD protein-based covid-19 vaccine zf2001 protects mice and nonhuman primates

**DOI:** 10.1080/22221751.2022.2056524

**Published:** 2022-04-11

**Authors:** Yaling An, Shihua Li, Xiyue Jin, Jian-bao Han, Kun Xu, Senyu Xu, Yuxuan Han, Chuanyu Liu, Tianyi Zheng, Mei Liu, Mi Yang, Tian-Zhang Song, Baoying Huang, Li Zhao, Wen Wang, Ruhan A, Yingjie Cheng, Changwei Wu, Enqi Huang, Shilong Yang, Gary Wong, Yuhai Bi, Changwen Ke, Wenjie Tan, Jinghua Yan, Yong-Tang Zheng, Lianpan Dai, George F. Gao

**Affiliations:** a Savaid Medical School, University of Chinese Academy of Sciences, Beijing, 101408, People’s Republic of China; b CAS Key Laboratory of Pathogenic Microbiology and Immunology, Institute of Microbiology, Chinese Academy of Sciences, Beijing, 100101, People’s Republic of China; c School of Life Sciences, University of Science and Technology of China, Hefei, Anhui, 230026, People’s Republic of China; d Kunming National High-Level Biosafety Research Center for Non-human Primates, Center for Biosafety Mega-Science, Kunming Institute of Zoology, Chinese Academy of Sciences, Kunming, 650107, People’s Republic of China; e Key Laboratory of Tropical Translational Medicine of Ministry of Education, School of Tropical Medicine and Laboratory Medicine, The First Affiliated Hospital, Hainan Medical University, Hainan, 571199, People’s Republic of China; f Research Network of Immunity and Health (RNIH), Beijing Institutes of Life Science, Chinese Academy of Sciences, Beijing, 100101, People’s Republic of China; g Laboratory of Animal Infectious Diseases, College of Animal Sciences and Veterinary Medicine, Guangxi University, Nanning, 530000, People’s Republic of China; h CAS Key Laboratory of Microbial Physiological and Metabolic Engineering, Institute of Microbiology, Chinese Academy of Sciences, Beijing, 100101, People’s Republic of China; i Key Laboratory of Animal Models and Human Disease Mechanism of the Chinese Academy of Sciences, Kunming Institute of Zoology, Chinese Academic of Sciences, Kunming, 650223, People’s Republic of China; j NHC Key Laboratory of Biosafety, National Institute for Viral Disease Control and Prevention, Chinese Center for Disease Control and Prevention, Beijing, 102206, People’s Republic of China; k Anhui Zhifei Longcom Biopharmaceutical Co. Ltd, Anhui, 230088, People’s Republic of China; l CAS Key Laboratory of Molecular Virology & Immunology, Institut Pasteur of Shanghai, Chinese Academy of Sciences, Shanghai 200031, People’s Republic of China; m Department of Microbiology-Infectiology and Immunology, Laval University, Quebec City G1, Canada; n CAS Center for Influenza Research and Early-Warning (CASCIRE), CAS-TWAS Center of Excellence for Emerging Infectious Diseases (CEEID), Chinese Academy of Sciences, Beijing, People’s Republic of China; o Guangdong Provincial Center for Disease Control and Prevention, Guangzhou 511430, People’s Republic of China; p Zhejiang University School of Medicine, Hangzhou 310058, People’s Republic of China

**Keywords:** Covid-19, subunit protein vaccine, zf2001, antibody, immune response

## Abstract

Safe, efficacious, and deployable vaccines are urgently needed to control COVID-19 in the large-scale vaccination campaigns. We report here the preclinical studies of an approved protein subunit vaccine against COVID-19, ZF2001, which contains tandem-repeat dimeric receptor-binding domain (RBD) protein with alum-based adjuvant. We assessed vaccine immunogenicity and efficacy in both mice and non-human primates (NHPs). ZF2001 induced high levels of RBD-binding and SARS-CoV-2 neutralizing antibody in both mice and non-human primates, and elicited balanced T_H_1/T_H_2 cellular responses in NHPs. Two doses of ZF2001 protected Ad-hACE2-transduced mice against SARS-CoV-2 infection, as detected by reduced viral RNA and relieved lung injuries. In NHPs, vaccination of either 25 μg or 50 μg ZF2001 prevented infection with SARS-CoV-2 in lung, trachea, and bronchi, with milder lung lesions. No evidence of disease enhancement was observed in both animal models. ZF2001 has been approved for emergency use in China, Uzbekistan, Indonesia, and Columbia. The high safety, immunogenicity, and protection efficacy in both mice and NHPs found in this preclinical study was consistent with the results in human clinical trials.

## Introduction

As of 21 Dec 2021, the coronavirus disease 2019 (COVID-19) pandemic caused by severe acute respiratory syndrome coronavirus 2 (SARS-CoV-2) infection has led to more than 274 million confirmed cases, with more than 5 million deaths [1,2]. Large-scale vaccinations are critical to fight against COVID-19 and control the pandemic. After 2 years of global efforts, a number of COVID-19 vaccines have been released the protection efficacies in Phase 3 clinical trials, and approved for use in many countries [3–5], including mRNA vaccines, adenovirus vectored vaccines, inactivated vaccines and protein subunit vaccines [6–18]. These approved vaccines mainly target whole virus or spike (S) protein [19]. Different vaccines with different mechanisms of action would benefit for the cost-effective countermeasure to systemically stop the COVID-19 pandemic. Protein subunit represents as an important avenue to develop COVID-19 vaccines, with 47 vaccine candidates in clinical phase documented in World Health Organization (WHO) until now [1].

In response to the pandemic, we developed a protein subunit vaccine targeting receptor-binding domain (RBD) [20,21]. SARS-CoV-2 RBD is located at the C-terminal domain of S1 subunit in S protein, and is responsible for the engagement of its cellular receptor human angiotensin-converting enzyme 2 (hACE2) [22]. RBD is an attractive coronavirus vaccine target because it focuses the antibody response to blocking receptor binding, therefore, poses low potential for antibody-dependent enhancement (ADE) risk [19,21,23]. To increase the immunogenicity, we designed a tandem-repeat dimeric RBD as the antigen for COVID-19 vaccine [21]. Compared with the traditional monomeric RBD, RBD-dimer significantly enhanced the SARS-CoV-2 neutralizing antibodies produced in mice [21]. We produced the RBD-dimer in Chinese hamster ovary (CHO) cell system, formulated with aluminium hydroxide as adjuvant. The resulting vaccine, ZF2001, showed safety, and immunogenicity in Phase 1 (NCT04445194, NCT04550351) and Phase 2 (NCT04466085) clinical trials [20]. Though the results are yet to be published, the ongoing multi-national Phase 3 clinical trial (NCT04646590) showed a clinical efficacy of 81.4% for ZF2001. ZF2001 had been approved for emergency use in China, Uzbekistan, Indonesia, and Columbia, and administered with more than 200 million doses in humans. Here, we report the preclinical studies of ZF2001 in both mouse and non-human primate (NHP) models.

## Materials and methods

### Mouse experiments

Speciﬁc pathogen-free (SPF) BALB/c and C57BL/6 mice were purchased from Beijing Vital River Laboratory Animal Technology Co., Ltd. (licenced by Charles River), and housed under SPF conditions in the laboratory animal facilities at Institute of Microbiology, Chinese Academy of Sciences (IMCAS). All mice were allowed free access to water and standard chow diet and provided with 12-hour light and dark cycle. All mice used in this study are in good health. The challenge studies with prototype SARS-CoV-2 were conducted under animal biosafety level 3 (ABSL3) facility in IMCAS. The mice experiments conducted in IMCAS were approved by the Committee on the Ethics of Animal Experiments of the IMCAS, and performed in compliance with the recommendations in the Guide for the Care and Use of Laboratory Animals of the IMCAS Ethics Committee.

Female BALB/c mice and female C57BL/6 mice were immunized intramuscularly (i.m) with two doses of ZF2001 (10 μg) or placebo, 21 days apart. Serum samples were collected after vaccination as indicated in figures legends.

For challenge experiment, C57BL/6 mice were intranasally (i.n.) transduced with 8 × 10^9^ vp of Ad5-hACE2 as a mouse model for SARS-CoV-2 infection [24,25]. Five days later, the transduced mice were infected with 5 × 10^5^ or 1 × 10^5^ TCID_50_ of SARS-CoV-2 (HB01 strain) via i.n. route. The mice were euthanized and necropsied 3 days or 5 days after challenge. Lung tissues were collected for virus titration and pathological examination. All mice experiments with SARS-CoV-2 challenge were conducted under animal biosafety level 3 (ABSL3) in IMCAS.

### Cynomolgus macaque experiments

Cynomolgus macaques of 3–4 years of age were all sourced from the Hainan Jingang Biotech Co., Ltd. They were kept in similar and independent primate cages, which facilitated communication between animals. The ABSL2 Laboratory has constant temperature (19–25°C) and humidity (50–69%), and circulates light (12 h light and 12 h dark), Central air conditioning centralized ventilation 8–10 times per hour, The Center of Safety Evaluation of Zhejiang Academy of Medical Sciences is fully certified by AAALAC (Association for Assessment and Accreditation of Laboratory Animal Care). The use of the experimental animals was approved by the Zhejiang Provincial Department of Science and Technology.

Cynomolgus macaques were divided into three groups (*n* = 10), with five females weighing 2.97–3.62 kg and five males weighing 2.95–3.67 kg in each group. Macaques were immunized with 25 μg or 50 μg ZF2001 vaccine via i.m. route at Week 0, 4, 8, and 10. Group receiving placebo was used as the control. Serum samples were collected for detection of SARS-CoV-2-specific IgG and neutralizing antibodies. Live SARS-CoV-2 neutralization assays were conducted under ABSL3 in IMCAS. The spleens of six macaques in each group were harvested to prepare splenocytes for detection of cytokines (IFN-γ, IL-2, and IL-4) by ELISPOT Assay at day 3 after the last vaccination. All policies and procedures utilized on the cynomolgus macaque experiments were in accordance with “Guide for the Care and Use of Laboratory Animals” and it has been approved by IACUC. Environmental enrichments were provided to all animals upon receipt in accordance with the center of safety evaluation IACUC policies.

### Rhesus macaque experiments

Rhesus macaques at 3–6 years of age were all sourced from the Kunming Primate Center of the Chinese Academy of Sciences. They were kept in similar and independent primate cages, which facilitated communication between animals. The ABSL3 Laboratory had constant temperature and humidity, and circulates light (12 h light and 12 h dark); the laboratory animals were monitored 24 h a day and were fed twice a day during the experiment. The staple foods were special pellets for primates and the green fodders were apples. Water was available ad libitum.

Rhesus macaques were divided into three groups (*n* = 3), with one female and two male monkeys in each group. Macaques were immunized with two doses of 25 μg or 50 μg ZF2001 via i.m. route, 21 days apart. Serum samples were collected for detection of SARS-CoV-2-specific IgG and neutralizing antibodies.

For challenge experiment, rhesus macaques were infected with 1 × 10^6^ TCID_50_ of SARS-CoV-2 (20SF107 strain) via intratracheal route. The experimental animals were anaesthetized by injection of Zoletil 50 (Virbac, France) into the thigh muscle. Euthanasia was performed on Day 7 after the challenge. Tissues from lungs (7 lobes with 4 sites in each lobe), trachea, left and right bronchus were collected for viral genomic RNA quantification and histopathology staining. The rhesus macaque experiment was approved by the animal ethics committee of Kunming Institute of Zoology, CAS. The selection of experimental animals and experimental operations had followed the guidelines and principles of the experimental animal ethics committee to ensure the welfare of experimental animals. The National Kunming High-Level Biosafety Primate Laboratory Center is accredited by the China National Accreditation Service for Conformity Assessment and performs related operations in accordance with the standard operating procedures approved by the Biosafety Committee of the Kunming Institute of Zoology, Chinese Academy of Sciences.

### Protein expression and purification

Monomeric RBD protein of SARS-CoV-2 used in ELISA assay was expressed and purified as previously described [21]. Briefly, the coding sequence for SARS-CoV-2 RBD (S protein 319-541, GISAID accession No. EPI_ISL_402119) was codon-optimized for mammalian cell expression and synthesized. For this construct, signal peptide sequence of MERS-CoV S protein (S protein residues 1–17) was added to the protein N terminus for protein secretion, and a hexa-His tag was added to the C terminus to facilitate further purification processes. The construct, which synthesized by GENEWIZ, China, was cloned into the pCAGGS vector and transiently transfected into HEK293T cells. After 3 days, the supernatant was collected and soluble protein was purified by Ni affinity chromatography using a HisTrap ^TM^ HP 5 ml column (GE Healthcare). The sample was further purified via gel filtration chromatography with HiLoad^®^ 16/600 Superdex^®^ 200 pg (GE Healthcare) in a buffer composed of 20 mM Tris-HCl (pH 8.0) and 150 mM NaCl.

### ELISA

For mice and rhesus macaque, ELISA plates (3590; Corning, USA) were coated overnight with 3 μg/ml of RBD protein in 0.05 M carbonate-bicarbonate buffer, pH 9.6, and blocked in 5% skim milk in PBS. Serum samples were serially diluted and added to each well. Plates were incubated with goat anti-mouse IgG-HRP antibody or goat anti-monkey IgG-HRP antibody and subsequently developed with 3,3′,5,5′-tetramethylbenzidine (TMB) substrate. Reactions were stopped with 2 M hydrochloric acid, and the absorbance was measured at 450 nm using a microplate reader (PerkinElmer, USA). The endpoint titre was defined as the highest reciprocal dilution of serum with absorbance greater than 2.5-fold of the background values. Antibody titre below the limit of detection was determined as half the limit of detection.

For cynomolgus macaques, ELISA plates were coated overnight with 1 μg/ml of RBD-dimer protein and blocked in 3% skim milk in PBST. Serum samples were serially diluted and added to each well. 10 μl serum from cynomolgus macaques of blank control group was mixed as one blank control serum and diluted as the same with an initial fold of vaccine group. The subsequent operations were as described above. Cut-off value was defined as 2.1-fold of OD_450_ value of the blank control serum. When OD_450 _> cut-off value, the serum was determined as positive. The endpoint titre was defined as the highest reciprocal dilution of serum with absorbance greater than 2.1-fold of the blank control serum value. When OD_450_ value of the serum to test_ _≤ cut-off value, the endpoint titre was zero.

### Live SARS-CoV-2 neutralization assay

The neutralizing activity of serum was assessed using a previously described SARS-CoV-2 neutralization assay [21]. Briefly, serum samples from mice and cynomolgus macaques were 4-fold serially diluted and mixed with the same volume of 100 TCID_50_ SARS-CoV-2 (IVDC HB-01 strain, GISAID accession No. EPI_ISL_402119), then incubated at 37°C for 1 h. Then, 100 μl virus-serum mixture was transferred to pre-plated Vero cells in 96-well plates. Inoculated plates were incubated at 37°C for an additional 72 h to monitor the cytopathic effect (CPE) microscopically. The neutralization titres were defined as the reciprocal of serum dilution required for 50% neutralization of viral infection. All the live virus neutralization assay of mouse and cynomolgus macaque serum samples was conducted under biosafety level 3 (BSL3) facility in IMCAS. The neutralizing activity of serum samples from rhesus macaques was assessed with 2020XN4276 strain of SARS-CoV-2 in the same method as mentioned before in Guangdong Provincial Center for Disease Control and Prevention [21].

### ELISPOT

To detect antigen-specific T lymphocyte responses, one ELISpot assay was performed. Briefly, mice spleens were harvested and splenocytes were isolated. Flat-bottom, 96-well plates which were precoated with 10 μg/ml anti-mouse IFN-γ Ab (BD Biosciences, USA) overnight at 4°C, were washed with sterile PBS and followingly blocked according to guidelines for Mouse IFN-γ ELISpot kit (BD). Splenocytes were added to the plate and then the peptide pool covering RBD of SARS-CoV-2 (2 μg/ml individual peptide) was added to the wells. Phytohemagglutinin (PHA) was added as a positive control. Cells without stimulation were employed as a negative control. After 24 h of incubation, the cells were removed, and the plates were processed in turn with biotinylated IFN-γ detection antibody, streptavidin-HRP conjugate, and substrate. When the coloured spots were intense enough to be visually observed, the development was stopped by thoroughly rinsing samples with deionized water. The numbers of the spots were determined using an automatic ELISPOT reader and image analysis software.

To detect antigen-specific T lymphocyte responses in macaques, pre-coated plates (mAb MT126L or mAb 2A91/2C95) and flat-bottom, 96-well plates, which were precoated with anti-monkey IL-4 Ab (U-Cytech) overnight at 4°C, were washed with sterile PBS and subsequently blocked according to guidelines for Monkey IFN-γ ELISPOT ^PLUS^ kit (MABTECH), IL-2 ELISPOT^PLUS^ kit (MABTECH) or IL-4 ELISPOT kit (U-Cytech). Splenocytes of cynomolgus macaques were added to the plate. RBD-dimer protein of SARS-CoV-2 was added to the wells for stimulation. Cells without stimulation were employed as a negative control. After 12–36 h of incubation, the cells were removed, and the plates were processed with biotinylated IFN-γ, IL-2 or IL-4 detection antibody, streptavidin-HRP conjugate, and substrate. When the coloured spots were intense enough to be observed, the development was stopped by thoroughly rinsing samples with deionized water. The numbers of the spots were determined using an automatic ELISPOT reader and image analysis software.

## ICS and flow cytometry

Mouse splenocytes were added to the plate (2 × 10^6^/well) and then stimulated with the peptide pool (2 μg/ml for individual peptide) for 5 h. The cells were incubated with GolgiStop (BD Biosciences, USA) for an additional 6 h at 37°C. Then, the cells were harvested and stained with anti-CD3 (BioLegend), anti-CD4 (BioLegend) and anti-CD8α (BioLegend) surface markers. The cells were subsequently fixed and permeabilized in permeabilizing buffer (BD Biosciences, USA) and stained with anti-mouse anti-IFN-γ (BioLegend), anti-TNF-α (BioLegend), anti-IL-2 (BioLegend), anti-IL-4 (BioLegend), and anti-IL-10 (BioLegend) antibodies. All fluorescent lymphocytes were gated on a FACSAria flow cytometer (BD Biosciences, USA).

### qRT-PCR for mice

The virus RNA quantitative experiments were performed as previously described [26], with some modifications. Mice lung tissues were weighed and homogenized. Virus genomic RNA was isolated from 50-μl supernatants of homogenized tissues using a nucleic acid extraction instrument MagMAX™ Express Magnetic Particle Processor (Applied Biosystems, USA). SARS-CoV-2-specific quantitative reverse transcription-PCR (qRT-PCR) assays were performed using a FastKing One Step Probe RT-qPCR kit (Tiangen Biotech, China) on a CFX96 Touch real-time PCR detection system (Bio-Rad, USA) according to the manufacturer’s protocol. Two sets of primers and probes were used to detect a region of the N gene of viral genome [27] and a region of E gene of subgenomic RNA (sgRNA) from SARS-CoV-2 [28], respectively, with sequences as follows: gRNA-N-F, GACCCCAAAATCAGCGAAAT; gRNA-N-R, TCTGGTTACTGCCAGTTGAATCTG; gRNA-N-probe, FAM-ACCCCGCATTACGTTTGGTGGACC-TAMRA (where FAM is 6-carboxyfluorescein, and TAMRA is 6-carboxytetramethylrhodamine); sgRNA-E-F, CGATCTCTTGTAGATCTGTTCTC; sgRNA-E-R, ATATTGCAGCAGTACGCACACA; sgRNA-E-probe, FAM-ACACTAGCCATCCTTACTGCGCTTCG-TAMRA. Viral loads were expressed on a log_10_ scale as viral copies/gram after calculation with a standard curve. Viral copy numbers below the limit of detection were set as half of the limit of detection.

### qRT-PCR for rhesus macaques

The total RNA of tissues from rhesus macaques were extracted with TRIzol reagent method (Thermo USA) [29], Viral RNA detection was detected with a probe one-step real-time quantitative PCR kit (TOYOBO, Japan). Previously reported primers (5′-GGGGAACTTCTCCTGCTAGAAT-3′, 5′-CAGACATTTTGCTCTCAAGCTG-3′) targeting SARS-CoV-2 N protein and probe (FAM-TTGCTGCTGCTTGACAGATT-TAMRA-3′) were used [30]. The dilution of each test run was referred to the standard (National Institute of Metrology, China), and the copy number of each sample was calculated.

### Histopathology analysis

Mice lung tissues were fixed in 4% paraformaldehyde, dehydrated, embedded in paraffin, and then sectioned. Tissue sections (slices 4 microns) were deparaffinized in xylene and stained with haematoxylin and eosin (H&E) for pathological examination, such as peribronchiolitis, interstitial pneumonitis, and alveolitis. All the rhesus macaque tissues were fixed in 4% paraformaldehyde for a minimum of seven days, then embedded in paraffin, sectioned (slices 4 microns) for H&E staining [31].

Pulmonary histopathology of lung tissue was scored based on the thickening of alveolar septa, pulmonary alveolar congestion, and inflammatory cell infiltration in alveoli and trachea. For thickening of alveola septa, no significant widened alveolar septa observed was scored 0; slightly widened alveolar septa with lesion range <25% was scored 1; moderate widened, fused, and consolidated alveolar septa with lesion range 25–50% was scored 2; severe widened, fused, consolidated alveolar septa with lesion range 50–75% was scored 3; extremely severe widened, fused, and consolidated alveolar septa with lesion range >75% was scored 4. For inflammatory cell infiltration and alveolar congestion, no lesion or disease variable were scored 0; mild/small lesions with lesion range or disease variable <25% were scored 1; moderate lesions with lesion range or disease variable 25–50% were scored 2; severe/multiple lesions with lesion range or disease variable 50–75% were scored 3; extremely severe/numerous lesions with lesion range or disease variable 50–75% were scored 4.

### Immunofluorescence

The paraffin tissue sections were deparaffinized with xylene, rehydrated through successive bathes of water, and incubated in 3% H_2_O_2_ at room temperature. Subsequently, the sections were blocked with BSA, incubated with primary antibody against SARS-CoV-2 nucleoprotein (Sino Biological) at 37°C and then incubated with goat anti-rabbit IgG (H + L) Alexa Fluor 488 antibody (Abways). DAPI (4′,6-diamidino-2-phenylindole) was also incubated with the sections, followed by detection using laser scanning confocal microscope (Leica).

### SPR

Protein interactions were tested through SPR analysis and the experiments were carried out at 25°C using a BIAcore 3000 machine with CM5 chips (GE Healthcare). All proteins for SPR analysis were exchanged to PBST (10 mM Na_2_HPO_4_; 2 mM KH_2_PO_4_, pH 7.4; 137 mM NaCl; 2.7 mM KCl; 0.005% Tween 20). SARS-CoV-2 RBD proteins were immobilized onto CM5 chips and analyzed for real-time binding by flowing through gradient concentrations of hACE2. The aforementioned RBD proteins were immobilized on the chip at about 1000 response units (RUs). The concentrations of hACE2 were 0, 0.78125, 1.5625, 3.125, 6.25, 12.5, 25, 50, 100, and 200 nM. After each cycle, the sensor surface was regenerated using 7 μl of 10 mM NaOH. Measurements from the reference flow cell (immobilized with BSA) were subtracted from experimental values. The binding kinetics were analyzed using 1:1 binding model with the software BIAevaluation Version 4.1 (GE Healthcare).

### Statistics analysis

Data were expressed as the means ± standard errors of the means (SEM) or geometric mean with 95% CI. For all analyses, *P-*values were analyzed with unpaired *t*-test or one-way ANOVA. Correlation analysis was conducted with Spearman correlation.

## Results

### Tandem-repeat RBD-dimer protein in ZF2001 vaccine

**Figure 1.**
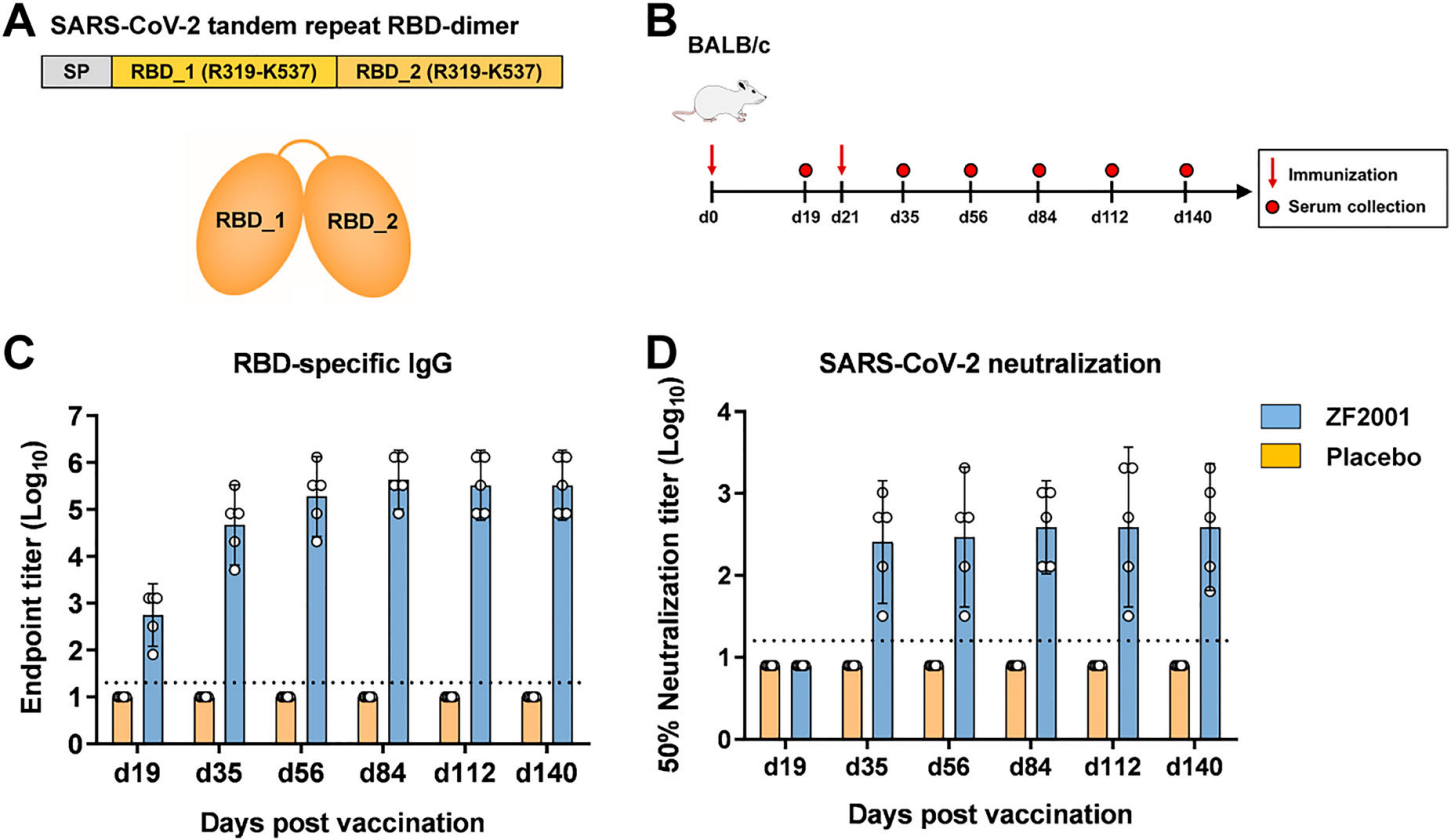
Humoral immune responses to ZF2001 vaccination in BALB/c mice. (A) schematic diagram of SARS-CoV-2 RBD dimer protein. Two copies of RBD from R319 to K537 are connected as tandem-repeat dimer. SP, signal peptide. (B) Time courses of ZF2001 vaccine immunization and sampling in groups of 6–8-week-old female BALB/c mice (*n* = 5) vaccinated with 10 μg ZF2001 or placebo. (C) Enzyme-linked immunosorbent assay (ELISA) show serum IgG against SARS-CoV-2 RBD. The dashed line indicates the limit of detection. Data are geometric mean with 95% CI. (D) SARS-CoV-2 (HB01 strain) neutralization assay shows the 50% neutralization titre. The dashed line indicates the limit of detection. Data are geometric mean with 95% CI.

The RBD construct for SARS-CoV-2 started at S protein residue R319 and stopped at residue K537. Two copies of RBD were connected as tandem repeat dimer. No purification tag was added (Figure 1(A)). The construct of RBD-dimer was transformed into clinical-grade CHO cell lines. Cell lines with highest antigen yields were selected for scaling-up antigen production in accordance with current Good Manufacturing Practice. The tandem-repeat RBD-dimer antigen was further purified and characterized [21]. The RBD-dimer bound to hACE2 with affinity similar to RBD-monomer, indicating the correct exposure of receptor-binding motif (Supplementary Figure 1). Stock solution was further formulated with aluminium hydroxide as adjuvant and put into vials as ZF2001 vaccine.

### ZF2001 vaccine immunogenicity in mice

To study the vaccine immunogenicity, groups of BALB/c mice (*n* = 5) were vaccinated with two doses of 10 μg ZF2001, 21 days apart. Mice receiving placebo (adjuvant-only) were used as the negative control. Serum samples were collected at different time points post vaccination to monitor the duration of antibody responses (Figure 1(B)). The first immunization of ZF2001 vaccine induced a geometric mean titre (GMT) of 557 for serological RBD-binding IgG (Day 19), and this titre was further increased to 47,051 after a second immunization (Day 35) (Figure 1(C)). For vaccine-induced SARS-CoV-2 neutralizing antibodies (NAbs), the GMT was 256 after two immunizations at Day 35 (Figure 1(D)). These titres remain high for each time point until Day 140, with a range of GMTs of RBD-binding IgG from 188,203 to 432,376 (Figure 1(C)), and a range of GMTs of SARS-CoV-2 NAb from 294 to 388 (Figure 1(D)), suggesting the durable humoral responses induced by ZF2001 in mice.

### ZF2001-elicited protection in Ad5-hACE2 transduced mice

**Figure 2.**
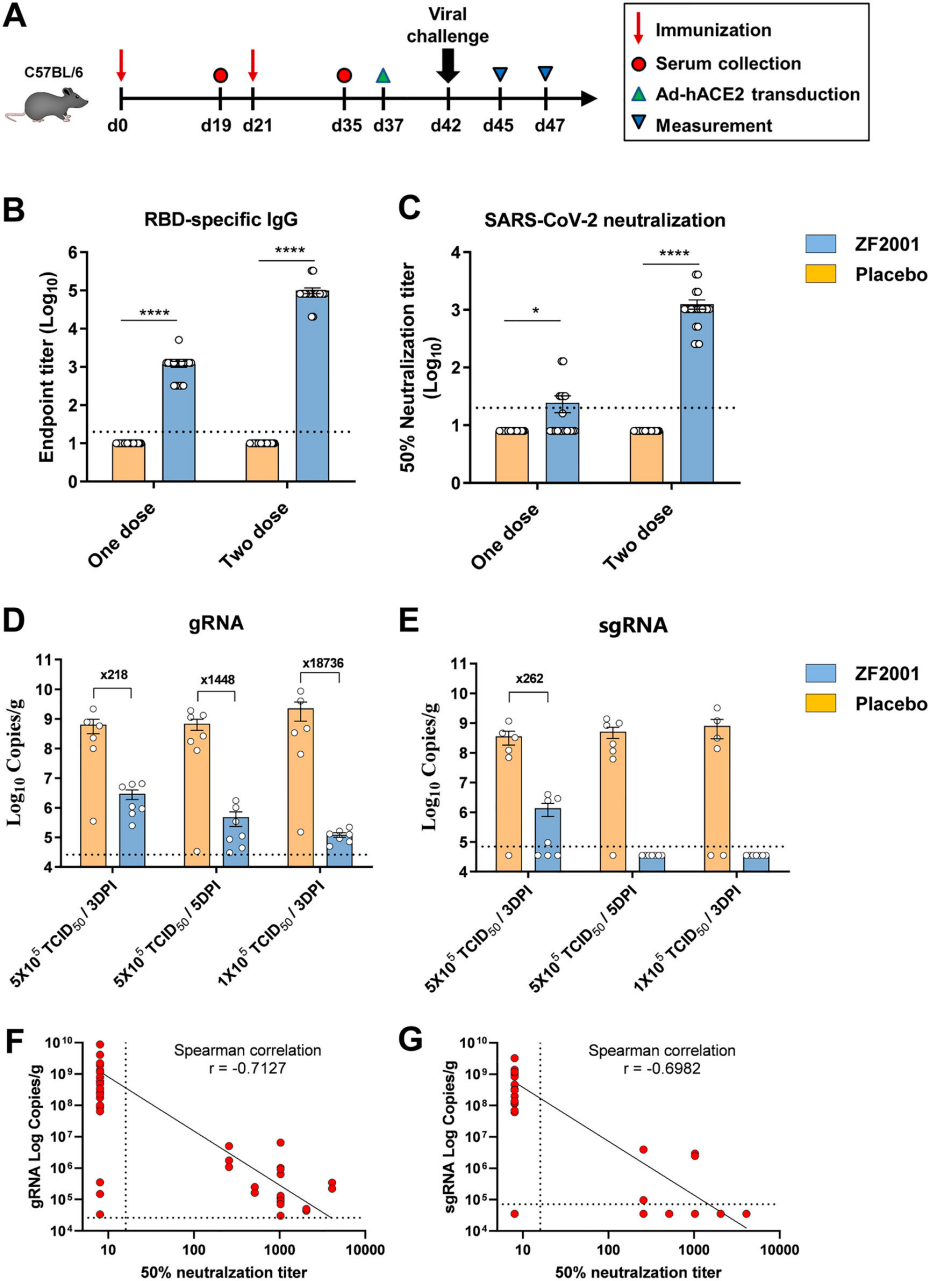
Protective efficacy of ZF2001 vaccine in mice. (A) Time course of immunization, sampling, viral challenge and measurement. Two groups of C57BL/6 mice (*n* = 21) were immunized with two doses of 10 μg ZF2001 vaccine or placebo, 3 weeks apart. Serum samples were collected after both one and two doses as indicated. Mice were then transduced with 8 × 10^9^ vp of Ad5-hACE2 via intranasal (i.n.) route. Each group of mice were further split into three groups (*n* = 6-7), with the former two groups infected with high-dose (5 × 10^5^ TCID_50_) SARS-CoV-2 and the latter group infected with low-dose (1 × 10^5^ TCID_50_) SARS-CoV-2. Lung tissues were harvested at either 3 or 5 DPI for the two groups with high-dose virus challenge and 3 DPI for the group with the low-dose virus challenge. (B) ELISA shows serum IgG against SARS-CoV-2 RBD. Data are geometric mean with 95% CI. *P*-values were analyzed with unpaired *t*-test (****, *P* < 0.0001). The dashed line indicates the limit of detection. (C) SARS-CoV-2 (HB01 strain) neutralization assay shows the 50% neutralization titre. Data are geometric mean with 95% CI. *P*-values were analyzed with unpaired *t*-test (*, *P* < 0.05; ****, *P* < 0.0001). The dashed line indicates the limit of detection. (D–E). SARS-CoV-2 titration from lung tissues by qRT-PCR probing virus gRNA (D) and sgRNA (E). Data are means ± SEM. The dashed lines indicate the limit of detection. (F–G) Protective correlation of NAb titre with SARS-CoV-2 gRNA (F) or sgRNA (G), calculated with Spearman correlation in GraphPad Prism 9.0.

**Figure 3.**
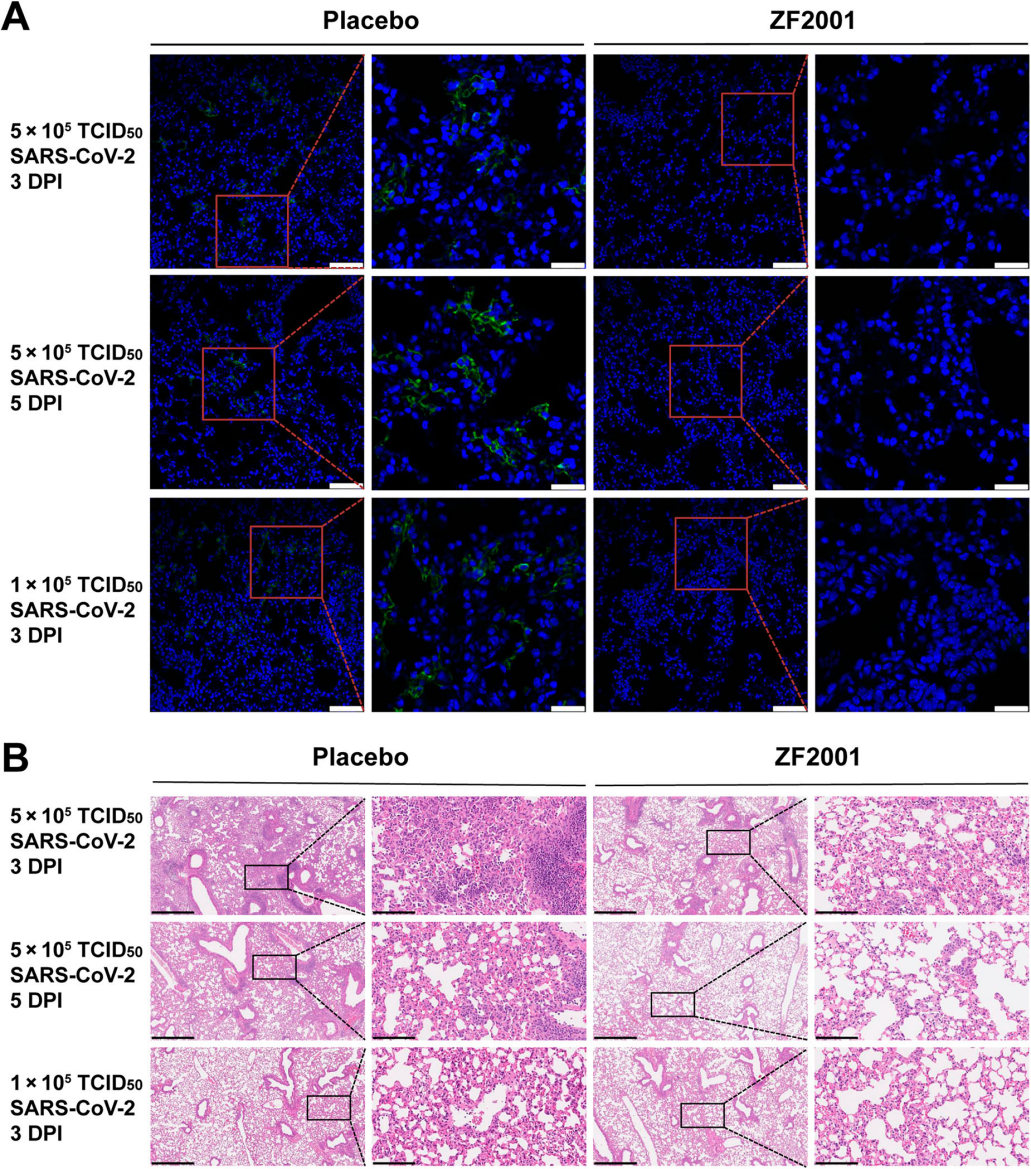
Immunofluorescence and histopathology analyses of lung tissues in mice. (A) Immunofluorescence analysis of lung tissue section stained with anti-SARS-CoV-2 nucleoprotein (N) antibody. Green: SARS-CoV-2 N protein; Blue: DAPI. Scale bar in low magniﬁcations images, 90 μm. Scale bar in high magniﬁcations images, 30 μm. (B) Typical histopathology images of lung tissues section shown by haematoxylin-eosin staining. Both low magniﬁcations and high magniﬁcations are shown, highlighted by boxes. Scale bar in low magniﬁcations images, 625 μm. Scale bar in high magniﬁcations images, 100 μm.

To study the protective efficacy of ZF2001 vaccine against SARS-CoV-2 infection, groups of C57BL/6 mice were immunized with two doses of 10 μg ZF2001, 21 days apart (Figure 2(A)). Mice receiving placebo were used as the control. Two doses of ZF2001 vaccine elicited high levels of both RBD-binding IgG (GMT, 81,920) and SARS-CoV-2 NAb (GMT, 959) (Figure 2(B,C)). These immunized mice were subsequently transduced via intranasal route with adenovirus expressing hACE2 as the SARS-CoV-2-sensitive animal model [24,25]. Five days later, these transduced mice were intranasally challenged with 5 × 10^5^ or 1 × 10^5^ 50% tissue culture infectious dose (TCID_50_) of SARS-CoV-2 (HB01 strain) [32,33]. Mice were euthanized and necropsied at 3 or 5 days post infection (DPI) to detect viral loads and exam pulmonary pathology. Encouragingly, for mice challenged with 5 × 10^5^ TCID_50_ SARS-CoV-2, the mean titres of viral genomic RNA (gRNA) per gram of lung in the placebo and ZF2001 group were 6.5 × 10^8^ and 3.0 × 10^6^, respectively, at 3 DPI with a 218-fold reduction, and were 7.0 × 10^8^ and 4.8 × 10^5^, respectively, at 5 DPI, with a 1,448-fold reduction (Figure 2(D)). For mice challenged with 1 × 10^5^ TCID_50_ SARS-CoV-2, the mean gRNA titres for the placebo and ZF2001 group were 2.3 × 10^9^ and 1.2 × 10^5^, respectively, with a 18,736-fold reduction of viral load in lung of ZF2001-vaccinated mice. The residual viral RNA in lung were probably derived from the high amount of input viruses. Therefore, we measured the magnitudes of subgenomic RNA (sgRNA) to quantify the infectious virus because sgRNA is generated in the infected cells during virus replication but is absent in virions [34]. In mice challenged with 5 × 10^5^ TCID_50_ SARS-CoV-2, the mean titres of viral sgRNA per gram of lung for placebo recipients were 3.6 × 10^8^ and 5.1 × 10^8^ at 3 DPI and 5 DPI, respectively. By contrast, this titre was reduced to 1.4 × 10^6^ at 3 DPI and undetectable at 5 DPI for ZF2001 vaccine recipients (Figure 2(E)). Accordingly, in mice challenged with 1 × 10^5^ TCID_50_ SARS-CoV-2, the mean sgRNA titre was 8.1 × 10^8^ for the placebo recipients, but was undetectable for the ZF2001 recipients, at 3 DPI (Figure 2(E)). The neutralizing antibody titres are negatively correlated with the gRNA and sgRNA, with Spearman correlation *r*-value of −0.7127 and −0.6982, respectively (Figure 2(F,G)). Consistent with this, immunofluorescence analysis of lung section stained with anti-SARS-CoV-2 NP antibody demonstrated the presence of viral protein in mice receiving placebo, but absence in mice receiving ZF2001 vaccine (Figure 3(A)). These results demonstrated that ZF2001 vaccine was protective against pulmonary SARS-CoV-2 infection.

To further assess the vaccine protection against SARS-CoV-2-induced lung damage, histopathological examination was performed. All lung tissue samples harvested from mice with placebo exhibited apparently moderated to severe viral pneumonia, characterized by thickened alveolar walls, vanishment of alveolar cavities, pulmonary vascular congestion, and diffuse inflammatory cell infiltration (Figure 3(B)). By contrast, markedly relieved histopathological changes were observed in lung tissues of mice vaccinated with ZF2001 vaccine (Figure 3(B)). This result demonstrated that ZF2001 protected against SARS-CoV-2-induced lung injury in mice, without observation of vaccine-enhanced diseases.

### ZF2001 vaccine immunogenicity in cynomolgus macaques

**Figure 4.**
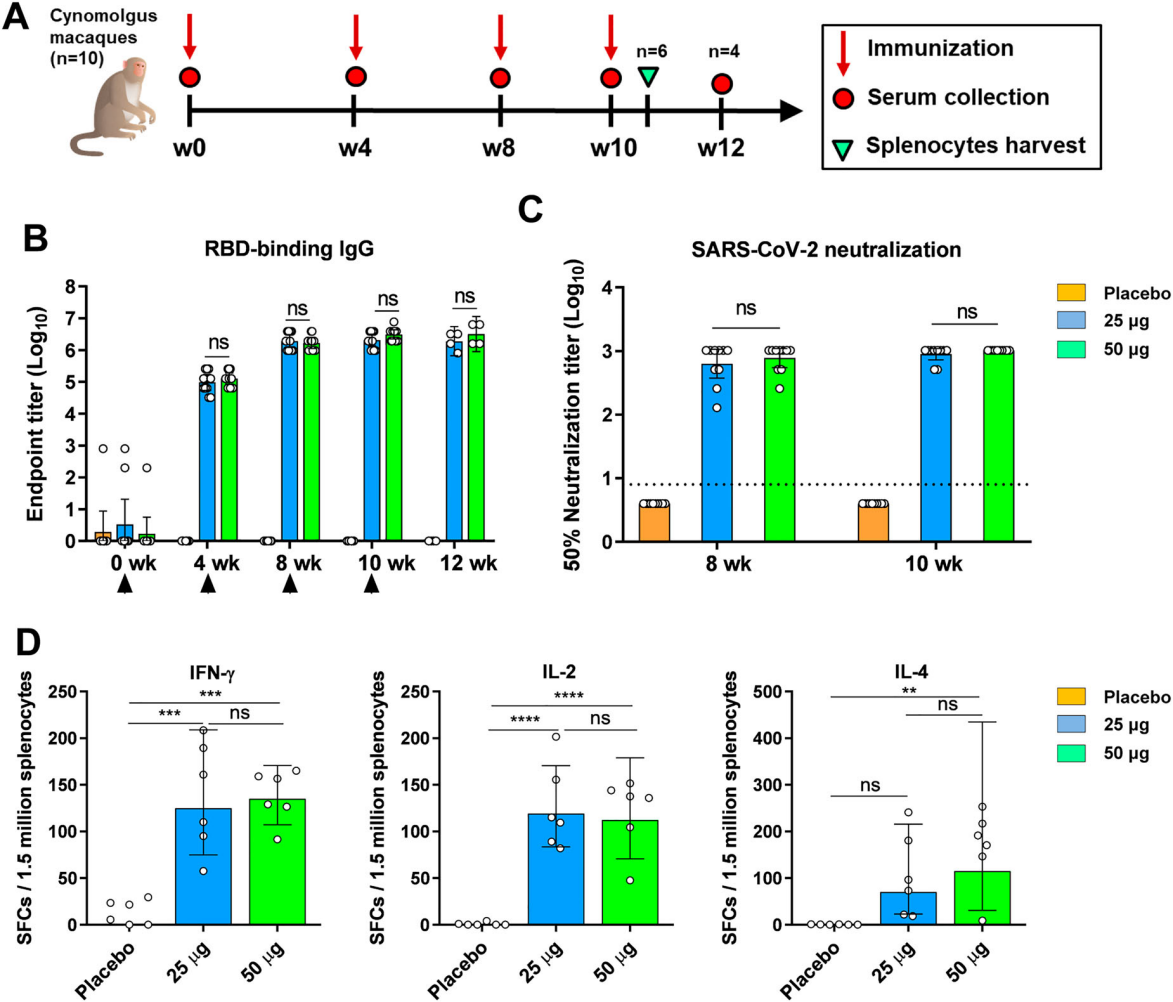
Humoral and cellular immune responses to ZF2001 vaccination in cynomolgus macaques. (A) Time course of immunization and sampling. Groups of cynomolgus macaques (*n* = 10) were immunized with four doses of placebo, the 25 μg vaccine and the 50 μg vaccine, respectively. Serum samples were collected at indicated time points post vaccination. Six macaques in each group were euthanized for spleen harvest at day 3 after the last vaccination. (B) ELISA shows serum IgG against SARS-CoV-2 RBD. Data are geometric mean with 95% CI. *P*-values were analyzed with unpaired t test (ns, not significant). (C) SARS-CoV-2 (HB01 strain) neutralization assay shows the 50% neutralization titre. Data are geometric mean with 95% CI. *P*-values were analyzed with unpaired t test (ns, not significant). The dashed line indicates the limit of detection. (D) Splenic IFN-γ, IL-2 and IL-4 ELISPOT responses to SARS-CoV-2 RBD antigen. SFCs: Spot-forming cells. Data are geometric mean with 95% CI. *P*-values were analyzed with one-way ANOVA (ns, not significant; **, *P* < 0.01; ***, *P* < 0.001; ****, *P* < 0.0001).

To further evaluate ZF2001 vaccine in NHPs, groups of healthy young cynomolgus macaques (*n* = 10) were immunized with four doses of 25 μg ZF2001, 50 μg ZF2001 or placebo intramuscularly at Week 0, 4, 8, and 10, respectively, to monitor the immunogenicity kinetics (Figure 4(A)). We found a single dose of ZF2001 vaccine elicit serological RBD-binding IgG GMTs up to ∼100,000 in both the 25 μg and 50 μg groups, and these titres were further enhanced to more than 1,000,000 after the second immunization (Figure 4(B)). The third and fourth boosts did not significantly increase the RBD-binding IgG GMTs (Figure 4(B)). For the NAb response, two immunizations of ZF2001 vaccine elicited SARS-CoV-2 neutralizing GMTs of 630 in the 25 μg group and 776 in the 50 μg group, respectively. The third immunization did not further enhance NAb titre (Figure 4(C)).

To evaluate the cellular immune responses in NHPs, 6 macaques in each group were euthanized to harvest spleen at 3 days after the last vaccination. Splenocytes were stimulated with RBD protein. ELISPOT assays were performed to detect T_H_1 (IFN-γ, IL-2) and T_H_2 (IL-4) cytokine production. We found both 25 μg or 50 μg ZF2001 induced substantial cellular responses, with the significantly enhanced and T_H_1/T_H_2 balanced cytokines production (Figure 4(D)).

Overall, ZF2001 induced robust immune responses in cynomolgus macaques. The cynomolgus macaques receiving 50 μg dose of vaccine did not show enhanced either humoral or cellular immune responses in comparison to those receiving 25 μg dose of vaccine.

### ZF2001-elicited protection in rhesus macaques

**Figure 5.**
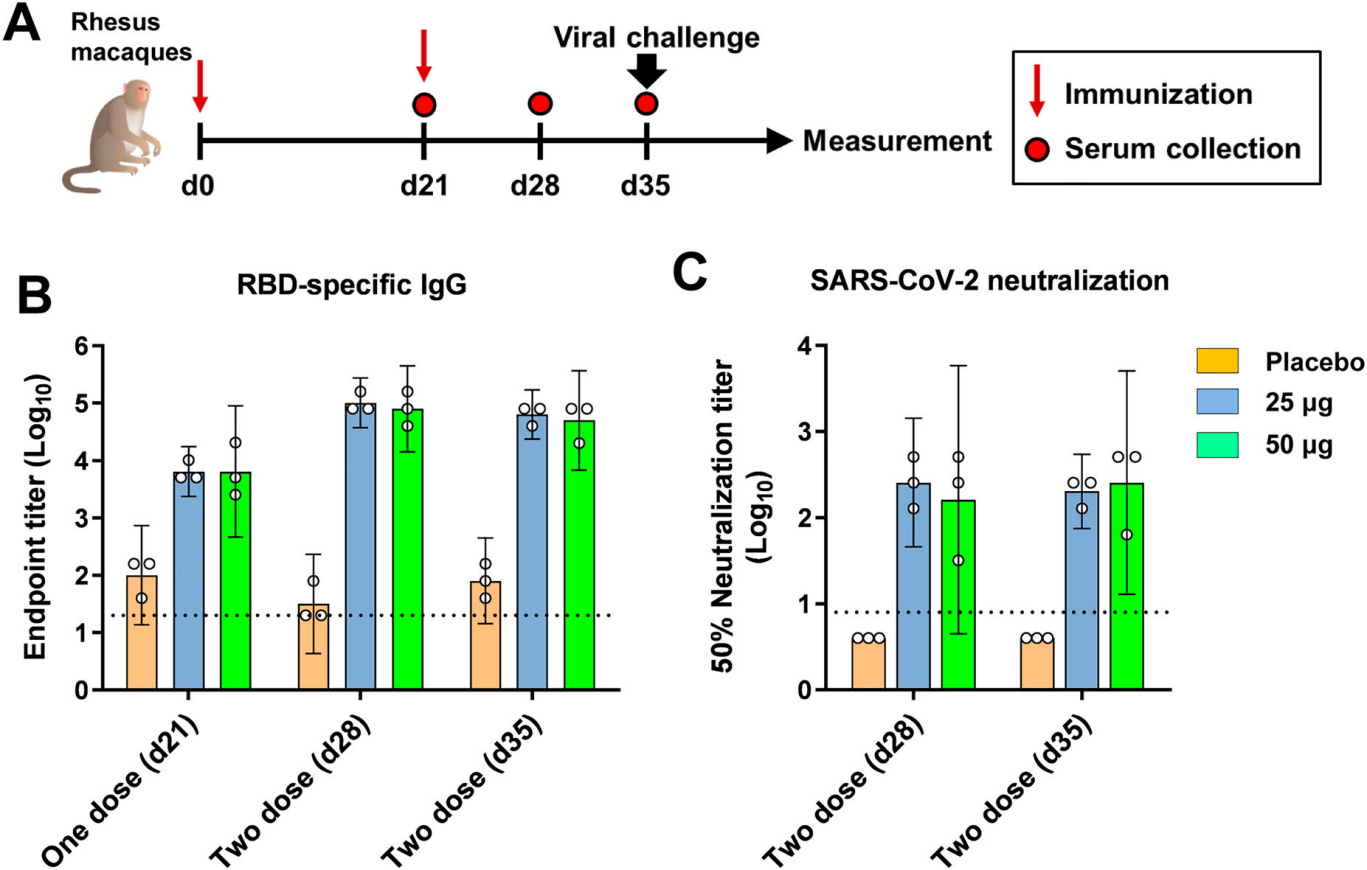
Humoral immune responses to ZF2001 vaccination in rhesus macaques. (A) Time course of immunization, sampling, viral challenge and measurement. Groups of rhesus macaques (*n* = 3) were immunized with two doses of placebo, the 25 μg vaccine and the 50 μg vaccine. Serum samples were collected at indicated time points post vaccination. At 14 post the second immunization, macaques were challenged with 1 × 10^6^ TCID_50_ SARS-CoV-2 (20SF107 strain) via intratracheal route. Macaques were euthanized at 7 DPI for tissue harvest. (B) ELISA shows serum IgG against SARS-CoV-2 RBD. Data are geometric mean with 95% CI. (C) SARS-CoV-2 neutralization assay shows the 50% neutralization titre. Data are geometric mean with 95% CI. The dashed line indicates the limit of detection.

To assess the protection efficacy, healthy young rhesus macaques (*n* = 3) were immunized with two doses of 25 μg or 50 μg vaccine intramuscularly, 21 days apart (Figure 5(A)). Macaques receiving placebo were used as the control. Both 25 μg and 50 μg ZF2001 vaccine elicited high levels of serological RBD-binding IgG and SARS-CoV-2 neutralizing antibody after two immunizations. For RBD-binding IgG, the GMTs reached 6451 for both the 25 μg and 50 μg groups after the first immunization (Figure 5(B)). After the second immunization, the GMTs further raised to 103,213 and 81,920 for the 25 μg and 50 μg groups, respectively, at Day 28 (Figure 5(B)). No significant increase was observed at Day 35, with the GMTs of 65,020 for the 25 μg group and 51,606 for the 50 μg group (Figure 5(B)). For SARS-CoV-2 NAbs, two doses of ZF2001 vaccine-elicited GMTs of 256 for the 25 μg group and 161 for the 50 μg group at Day 28 (Figure 5(C)). No significant increase was observed at Day 35, with the GMTs of 203 for the 25 μg group and 256 for the 50 μg group (Figure 5(C)). The 50 μg group did not show enhanced immunogenicity compared to the 25 μg group in rhesus macaques.

**Figure 6.**
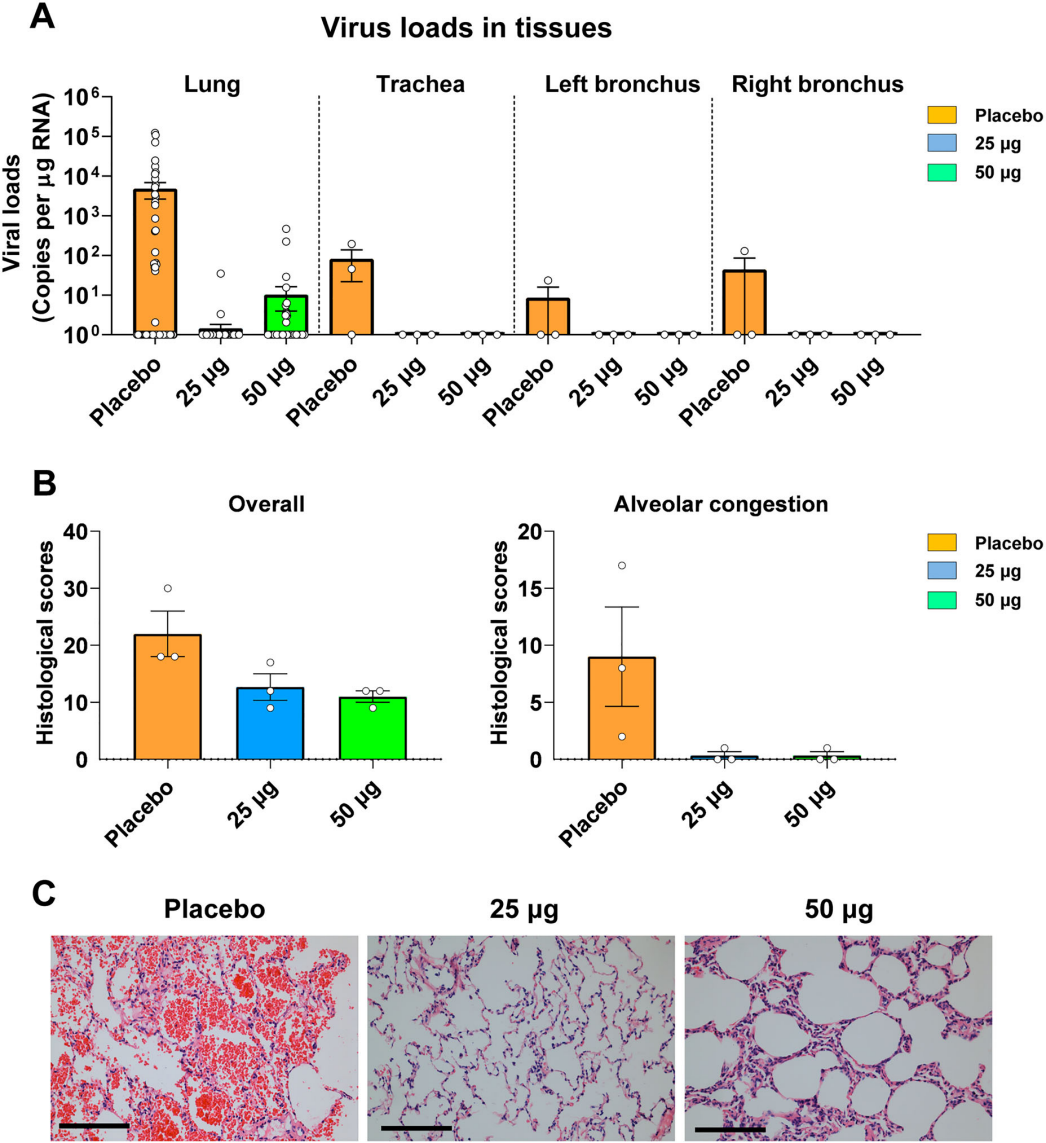
Viral loads and clinical signs in rhesus macaques challenged with SARS-CoV-2 after vaccination. (A) SARS-CoV-2 titration from lung tissues by qRT-PCR probing virus gRNA. Tissue samples: 7 lung lobes (4 sites for each lobe) of three macaques in each group (84 samples per group); tracheas of three macaques in each group; left and right bronchi of three macaques in each group. (B) Histopathology scores of overall lung lesions and pulmonary alveolar congestion. (C) Typical histopathology images of lung tissues section shown by haematoxylin-eosin staining. Scale bar, 100 μm.

These macaques were challenged with 1 × 10^6^ TCID_50_ SARS-CoV-2 intratracheally at Day 35 after priming (Figure 5(A)). Macaques were euthanized at 7 DPI. Tissues from 7 lung lobes (4 sites in each lobe), trachea, left and right bronchus were collected and quantified for viral gRNA. As expected, substantial virus loads could be detected in lung lobes (average 4748 copies/μg gRNA). By contrast, animals receiving either 25 μg or 50 μg ZF2001 vaccine exhibited significantly reduced virus loads in lung lobes, with average 1 and 10 copies/μg gRNA for 25 μg and 50 μg group, respectively. In addition, viral gRNA was detected in trachea and bronchi from 1 or 2 macaques receiving placebo, but was undetectable in all ZF2001-vaccinated macaques (Figure 6(A)).

To further assess the protection efficacy in lung, pulmonary histopathology of each macaque was scored based on the thickening of alveolar septa, pulmonary alveolar congestion, and inflammatory cell infiltration in alveoli and trachea. Overall, high scores of lung lesions were found in control animals, but were dramatically reduced in ZF2001-vaccinated animals (Figure 6(B)). In particular, all three control animals showed severe pulmonary alveolar congestion (Figure 6(C)). By contrast, macaques receiving either 25 μg or 50 μg vaccine showed almost no alveolar congestion (Figure 6(C)). The typical images of tissue haematoxylin-eosin (H&E) staining were shown in Figure 6(C). In addition, we did not observe vaccine-enhanced diseases in all these macaques.

## Discussion

ZF2001 is a tandem-repeat dimeric RBD protein COVID-19 vaccine, which has been approved for emergency use in China, Uzbekistan, Indonesia, and Columbia since March 2021, with more than 200 million doses administered globally. The Phase 3 clinical trials (NCT04646590) showed ZF2001 conferred more than 80% of protection efficacy against symptomatic COVID-19 [35]. Here, we report its preclinical results in animal models of both mice and NHPs. ZF2001 vaccine was highly immunogenic in both mouse and NHP models, with high neutralizing GMTs against SARS-CoV-2 persisting for a long period. Two shots of vaccine protected both hACE2-transduced mice and NHPs against SARS-CoV-2 infection and pneumonia. No evidence of vaccine-enhanced diseases was found in both models. Compared with the other two protein subunit vaccines advanced in Phase 3 or 2/3 clinical trials (NVX-CoV2373 and SCB-2019) using full-length S protein as antigen, ZF2001 vaccine is an RBD-based protein vaccine aiming to focus immune responses in receptor-binding blocking, with a unique design of dimer conformation for better stability and immunogenicity [21]. Besides, ZF2001 vaccine uses a traditional alum-based adjuvant which has long safety profiles in humans, rather than the relatively new adjuvants used in NVX-CoV2373 (Matrix-M1) and SCB-2019 (AS03 or CpG1018 + Alum) [36,37]. In addition, ZF2001 is stable in 2–8°C for storage, which will make it easier and more convenient for transportation/storage in low-resource countries. Moreover, ZF2001 can be manufactured in large scale due to its high antigen yields [21], which highlights the advantage of vaccine deployment to meet the global vaccine demands. Therefore, the encouraging preclinical results suggest that ZF2001 vaccine with different vaccine target, different adjuvant, and different mechanism of action would diversify the current vaccine pipelines.

In NHPs models, the higher vaccine dose (50 μg) did not show a superior immune response or better protection compared to the lower vaccine dose (25 μg). We speculate that 25 μg dose has already reached or is near to a saturated dosage to stimulate the immune system. Larger dose may not further increase the immunogenicity. On the contrary, sometimes larger dose may decrease the protection as reported for an adenovirus-based vaccine AZD1222 [38]. A similar immunological trend was also observed in Phase 1 and 2 clinical trials [20].

SARS-CoV-2 RBD-dimer immunogen was also used in other platforms. Different platforms have their own advantages. We previously reported a chimpanzee adenovirus virus vectored vaccine (AdC7) expressing RBD-dimer (AdC7-RBD-tr2) as vaccine [39]. By an indirect comparison, ZF2001 appears to elicit higher binding and neutralizing antibodies after two doses of vaccination in mice in comparison to two doses of AdC7-RBD-tr2. Instead, the adenovirus vectored vaccine appears to elicit more pronounced T cell responses after two doses of vaccination in mice in comparison to ZF2001 (supplementary Figure 3). Both forms of vaccine achieved good protections against SARS-CoV-2 challenge in mice, with undetectable virus sgRNA. However, a head-to-head comparison of these two vaccines is required for a more accurate conclusion.

The cynomolgus macaques used here for immunogenicity were also designed for repetitive dose toxicity testing. Therefore, four doses of vaccinations were performed and gave us a chance to investigate the kinetics of vaccine-elicited immune responses. The results showed two doses of ZF2001 were already sufficient to maximize the binding and neutralization antibodies in cynomolgus macaques. Two-dose of ZF2001 also elicited high levels of humoral responses in BALB/c mice, C57BL/6 mice, and rhesus macaques. However, in our phase 1 and 2 clinical trial in humans [20], the third dose was required to further enhance the titres of neutralizing antibodies (5–6 folds of the titre of two doses vaccination). Therefore, a three-dose regimen of ZF2001 was used in phase 3 trial and the real-world practice. This difference of immunogenicity between humans and animals needs to be further investigated.

In sum, this preclinical results in NHPs support the use of 25 μg vaccine dose to the ongoing phase 3 trial and vaccination practice in the real world.

## Data and materials availability

All data associated with this study are available from the corresponding author upon reasonable request.

## Supplementary Material

Supplemental Material
