# Supplementary material for: A tandem-repeat dimeric RBD protein-based covid-19 vaccine zf2001 protects mice and nonhuman primates
Source: Emerg Microbes Infect. 2022 Apr 11;11(1):1058–71. doi: 10.1080/22221751.2022.2056524 (PMC9009945; doi:10.1080/22221751.2022.2056524)
Supplement: Supplemental Material [file TEMI_A_2056524_SM8985.docx]

**Supplemental material**

**A tandem-repeat dimeric RBD protein-based COVID-19 vaccine ZF2001 protects mice and nonhuman primates**

Yaling An^#^, Shihua Li^#^, Xiyue Jin^#^, Jian-bao Han^#^, Kun Xu^#^, Senyu Xu, Yuxuan Han, Chuanyu Liu, Tianyi Zheng, Mei Liu, Mi Yang, Tian-zhang Song, Baoying Huang, Li Zhao, Wen Wang, Ruhan A, Yingjie Cheng, Changwei Wu, Enqi Huang, Shilong Yang, Gary Wong, Yuhai Bi, Changwen Ke, Wenjie Tan*, Jinghua Yan*, Yong-tang Zheng*, Lianpan Dai*, George F. Gao*

*Corresponding author. Email: gaof@im.ac.cn (G.F.G.), dailp@im.ac.cn (L.D.), zhengyt@mail.kiz.accn (Y.Z.), yanjh@im.ac.cn (J.Y.), tanwj@ivdc.chinacdc.cn (W.T.)

**Supplementary Figure 1**


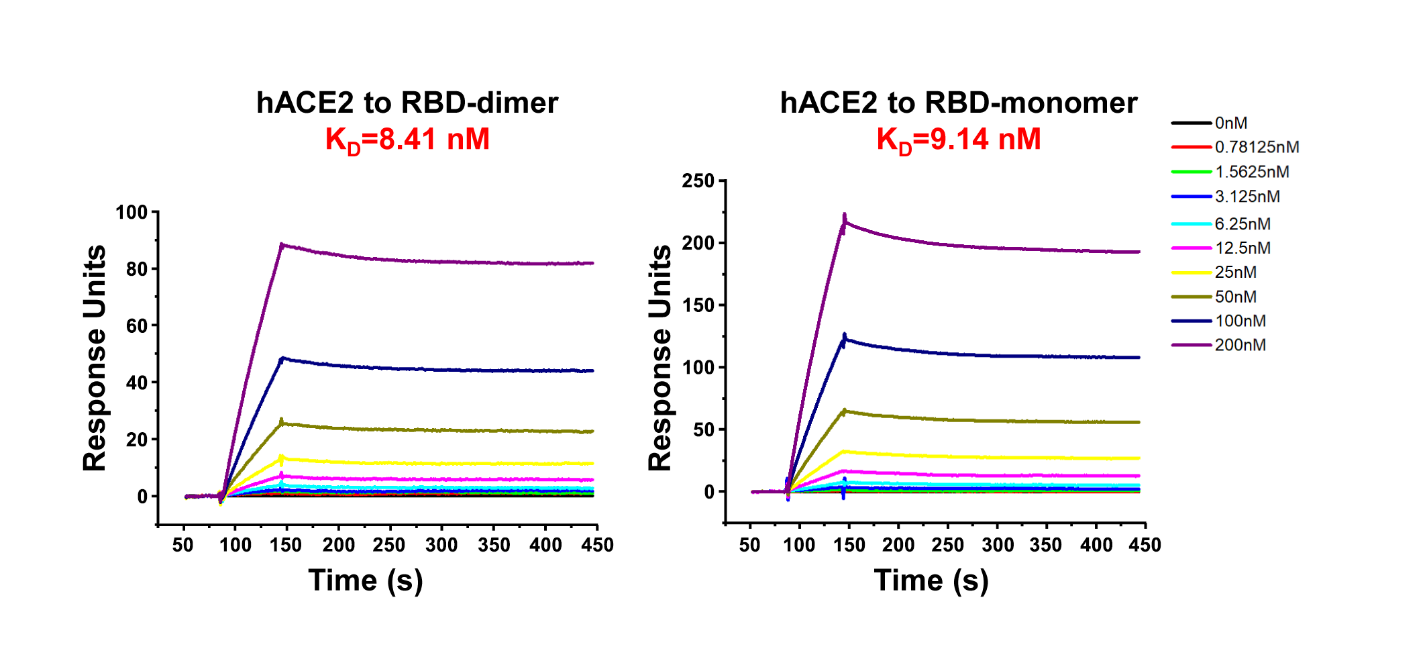


Supplementary Figure 1. Representative BIAcore diagrams of RBD-dimer and RBD-monomer bound to hACE2 protein. The K_D_ value was calculated by the software BIAevaluation Version 4.1 (GE Healthcare).

**Supplementary Figure 2**


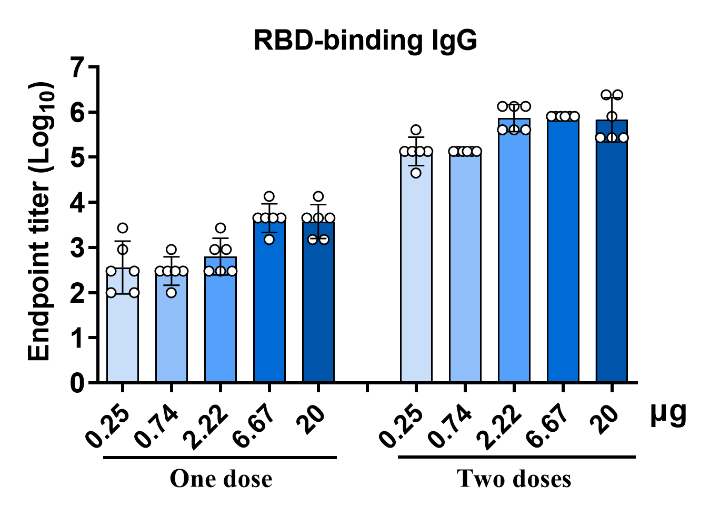


Supplementary Figure 2. RBD-binding antibody elicited by one/two injections of RBD-dimer antigen with escalation doses.

**Supplementary Figure 3**


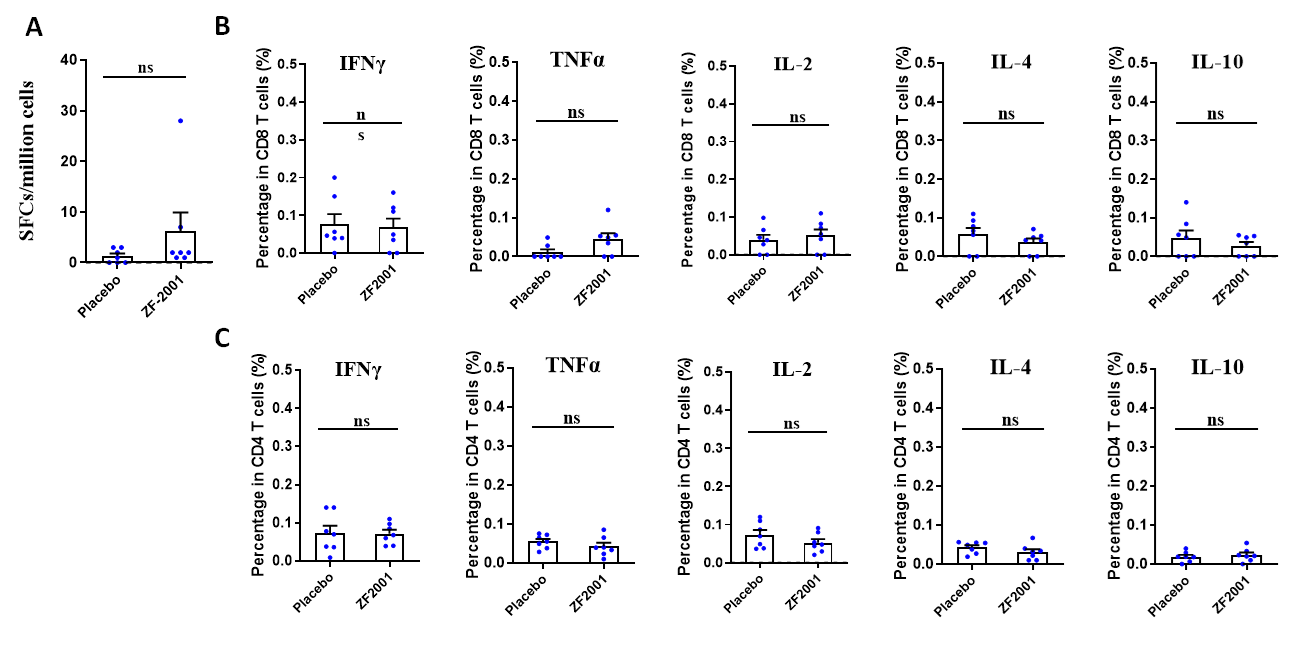


Supplementary Figure 3. Cellular immune responses to ZF2001 vaccination in mice. BALB/c mice were immunized with 10 μg ZF2001 or placebo. (A) The IFN-γ secretion of splenocytes after SARS-CoV-2 S antigen stimulation by ELISpot assays. (B) The IFN-γ, TNFα, IL-2, IL-4, and IL-10 producing CD8+ T cells responses to SARS-CoV-2 S antigen by ICS assays. (C) The quantification of the frequency of IFNγ, TNFα, IL-2, IL-4, and IL-10 producing CD4+ T cells responses to SARS-CoV-2 S antigen by ICS assays.Data are means ± SEM. Pvalues were analysed with t-test (ns, P>0.05;*,P<0.05;**,P<0.01;***,P<0.001;****,P<0.0001).
